# Patient experiences, attitudes, and profiles regarding artificial intelligence in rheumatology: a German national cross-sectional survey study

**DOI:** 10.1007/s00296-025-06023-x

**Published:** 2025-11-10

**Authors:** Hannah Labinsky, Philipp Klemm, Lukas Graalmann, Thea Thiele, Johannes Hornig, Daniel Fink, Harriet Morf, Johanna Mucke, Uta Kiltz, Ann-Christin Pecher, Alexander Pfeil, Corinna Elling-Audersch, Gerlinde Bendzuck, Martin Krusche, Axel J. Hueber, Johannes Knitza

**Affiliations:** 1 Department of Internal Medicine 2, Rheumatology/Clinical Immunology, University Hospital Würzburg, Würzburg, Germany; 2 Department of Rheumatology, Clinical Immunology, Osteology and Physical Medicine, Justus-Liebig University Giessen, Campus Kerckhoff, Bad Nauheim, Germany; 3 Department of Rheumatology and Immunology, Hannover Medical School, Hannover, Germany; 4 Rheumapraxis an der Hase, Osnabrück, Germany; 5 Rheumazentrum Mittelhessen, Bad Endbach, Germany; 6 Department of Medicine 3 - Rheumatology and Immunology, Friedrich- Alexander-Universität Erlangen-Nürnberg and Uniklinikum Erlangen, Erlangen, Germany; 7 Rheumazentrum Ruhrgebiet, Herne, Germany; 8 Department of Internal Medicine II, Hematology, Oncology, Clinical Immunology, and Rheumatology, University Hospital Tübingen, Tübingen, Germany; 9 Department of Internal Medicine III, Jena University Hospital-Friedrich Schiller University Jena, Jena, Germany; 10 German League against Rheumatism, Bonn, Germany; 11 Division of Rheumatology and Systemic Inflammatory Diseases, Department of Medicine, University Medical Center Hamburg-Eppendorf, Hamburg, Germany; 12 Department Internal Medicine 5, Division of Rheumatology, Klinikum Nuremberg, Paracelsus Medical University, Nuremberg, Germany; 13 Institute for Digital Medicine, School of Medicine, Philipps-Universität Marburg, Marburg, Germany

**Keywords:** Surveys and questionnaires, ChatGPT, Large language models, Artifical intelligence, Patient self-management

## Abstract

While artificial intelligence (AI) is gaining attention in rheumatology, little is known about patient perspectives. This study addresses this gap by examining patients’ experiences and attitudes toward AI. A nationwide, cross-sectional, web-based survey was conducted between March and May 2025 among adult patients with rheumatic diseases in Germany. Data were analyzed descriptively and with cluster analysis. A total of 778 patients completed the survey (70.4% female, mean age 51.3 years). The most common diagnosis was rheumatoid arthritis (31.7%). While 26.8% reported current AI use for health-related purposes, 57.8% expressed interest in using it. Patients were particularly interested in AI-based symptom checkers (64.3%), therapy recommendations (50.6%), and chatbots for medical inquiries (44.5%). 57.6% of patients indicated that they would welcome their rheumatologists using AI-based clinical suppport. The most frequently cited benefits of AI included improved information access (63.5%) and faster diagnosis (57.7%), while concerns centered on faulty AI (74.3%) and reduced human interaction (59.6%). Cluster analysis identified three distinct patient profiles: ‘AI-savvy’ (41.4%), ‘AI-pragmatic’ (44.8%), and ‘AI-skeptical’ (13.8%). Cluster membership was significantly associated with age and education, with younger patients more often belonging to the ‘AI-savvy’ group. Patients with rheumatic diseases showed substantial interest in AI-supported care, although actual use in medical contexts remained limited. Age and education differences highlight the need for tailored implementation strategies to ensure equitable and patient-centered adoption of AI in rheumatology.

**Supplementary Information:**

The online version contains supplementary material available at 10.1007/s00296-025-06023-x.

## Introduction

Artificial intelligence (AI) has the potential to transform all stages of the patient journey [1]. Especially large language models (LLMs), such as ChatGPT, may accelerate the implementation of AI in rheumatology by rapidly transforming the traditional patient–physician relationship into a digital health triad, complemented by AI. Recent studies have demonstrated that LLMs can match rheumatologists in diagnostic accuracy [2] and, in some cases, outperform approved and established medical diagnostic support systems [3]. They are increasingly capable of providing safe treatment recommendations across a broad spectrum of rheumatic diseases [4]. Notably, for patient questions related to general medicine and also systemic lupus erythematosus (SLE), LLM-generated answers were rated as more accurate, higher in quality, and more empathetic than those provided by human experts [5, 6]. These initial findings underscore the transformative potential of LLMs in augmenting rheumatology care.

However, the successful integration of AI into routine care depends not only on its technical performance but also on patient acceptance, trust, and willingness to engage with these technologies. While the technical capabilities of AI in rheumatology have been increasingly studied, these evaluations have typically included rheumatologists [7] and relied on their assessments [8]. To our knowledge, few studies have examined patient perspectives on AI specifically within rheumatology.

This study aimed to address this critical gap by conducting a nationwide web-based survey to explore rheumatic patients’ experiences, attitudes, and expectations toward AI. The results offer new insights to guide the development of AI tools in rheumatology and to shape healthcare policies and practices that promote patient-centered and responsible AI use.

## Methods

An initial draft of the questionnaire in German was collaboratively developed by four rheumatologists (JK, HL, MK, AH) based on a comprehensive literature review. To ensure content and face validity, the draft was additionally reviewed by two patient research partners (GB, CEA) affiliated with the German League Against Rheumatism. Based on their feedback, both the content and wording of the survey were revised.

The final questionnaire included 18 questions (English translation, see Supplementary File 1). Participation took approximately 15 min per participant. The questionnaire collected demographic data (5 questions) including age, sex, education level, and type of rheumatology treatment center. Participants were also asked to indicate their specific rheumatic disease. In addition, the survey assessed prior experiences with AI in a medical context (3 questions), as well as attitudes regarding its use in healthcare, including interest, willingness to use, and potential concerns (10 questions).

An anonymized cross-sectional web-based study was conducted via REDCap (Research Electronic Data Capture; Vanderbilt University), hosted by the University of Marburg, Germany, and was accessible between March 25 and May 30, 2025. Adults currently receiving care from a rheumatologist were eligible to participate. The survey was distributed through QR codes in German university and non-university rheumatology clinics and promoted by the German League Against Rheumatism via its newsletter and website. The Ethics Committee of Philipps-University Marburg confirmed that formal ethical approval was not required for this anonymous survey study (reference: 25–89 ANZ). No formal sample size calculation was performed, as this study followed an exploratory design.

Results were reported according to recommendations by Zimba and Gasparyan [9]. Parametric statistical methods were applied based on the central limit theorem. Numerical variables were summarized as means and standard deviations, while categorical variables were described using absolute and relative frequencies (percentages). Group differences in survey responses by clinical and demographic characteristics were analyzed using t-tests with Cohen’s d and one-way analysis of variance (ANOVA) with Eta squared following Tukey’s HSD post hoc tests.

To identify distinct patient profiles based on patients’ responses to AI-related items, an unsupervised cluster analysis was conducted. Only complete data sets (*N* = 739) were included. Prior to clustering, relevant variables were standardized. The data for the variables AI usage, AI-supported second opinion, providing data for AI research, self-management, and disease monitoring were recoded so that higher values indicated stronger usage or more positive assessments. Scores for individual dimensions with multiple sub-dimensions were calculated by taking the row-wise mean of the relevant sub-dimension variables (potential AI applications, perceived advantages, facilitators and barriers). Dimensionality reduction was performed using Principal Component Analysis (PCA) to explore the data structure and visualize group separation. K-means clustering was applied, and the optimal number of clusters was determined using the elbow method [10]. Differences between clusters were assessed using one-way ANOVA followed by Tukey’s HSD post hoc tests. Associations between cluster membership and demographic or clinical variables were examined using Fisher’s exact tests (pairwise, where appropriate). In addition to p-values, effect sizes were calculated to quantify the magnitude of observed differences. Eta squared (η²) was reported for continuous outcomes such as AI interest, perceived usefulness, and AI usage. Effect sizes (η²) were interpreted as small (≈ 0.01), medium (≈ 0.06), and large (≈ 0.14). Cramér’s V was used to measure the strength of association between categorical variables (e.g., sex, age group, education, healthcare setting, and disease) and cluster membership. All analyses and figures were generated in R (version 4.5.0; RStudio 2025.05.0) using the packages FactoMineR, ggplot2, and cluster. Figures were generated in R using *ggplot2* and *factoextra*.

For the Sankey diagram, categorical data were preprocessed in R and then exported to the online tool SankeyMATIC (https://sankeymatic.com/) for visualization.

## Results

### Patient Demographics

A total of 778 patients completed the survey (Table 1). The majority were female (70.4%), mean age (SD) was 51.3 (14.2) years. Regarding educational background, 30.8% had obtained a university degree, 19.2% had completed vocational training, and 19.0% held a secondary school certificate. Most participants were recruited from university hospitals (38.7%) or outpatient rheumatology practices (38.2%). Rheumatoid arthritis was the most frequently reported diagnosis (31.7%), followed by psoriatic arthritis (12.7%), axial spondyloarthritis (10.2%), SLE (10.0%), and vasculitis (7.3%).

**Table 1:** Demographics

Category	Subcategory	Total (*n* = 778)	Percentage (%)
Sex	Female	548	70.4
	Male	229	29.4
	Other	0	0
	Unknown	1	0.1
Age, years	18–39	172	22.1
	40–59	359	46.2
	≥ 60	245	31.7
	Unknown	1	0.1
	Mean (SD)	51.3 (14.2)	---
Education level	No school-leaving certificate	2	0.3
	Basic secondary school certificate	41	5.3
	Secondary school certificate	148	19.0
	German A-level equivalent	109	14.0
	Completed vocational training	149	19.2
	Completed technical school	79	10.2
	Completed university degree	240	30.8
	Other	10	1.3
Treating center	University hospital	301	38.7
	General hospital	178	22.9
	Outpatient practice	297	38.2
	Unknown	2	0.3
Disease	Rheumatoid Arthritis	247	31.7
	Psoriatic Arthritis	99	12.7
	Axial Spondyloarthritis	79	10.2
	Systemic Lupus Erythematosus	78	10.0
	Vasculitis	57	7.3
	Myositis	53	6.8
	Other connective tissue disease	52	6.7
	Osteoarthritis	18	2.3
	Fibromyalgia	17	2.2
	Other	68	8.7
	Unknown	10	1.3

### Patient Use and Attitudes towards AI in Rheumatology

**Fig. 1:**
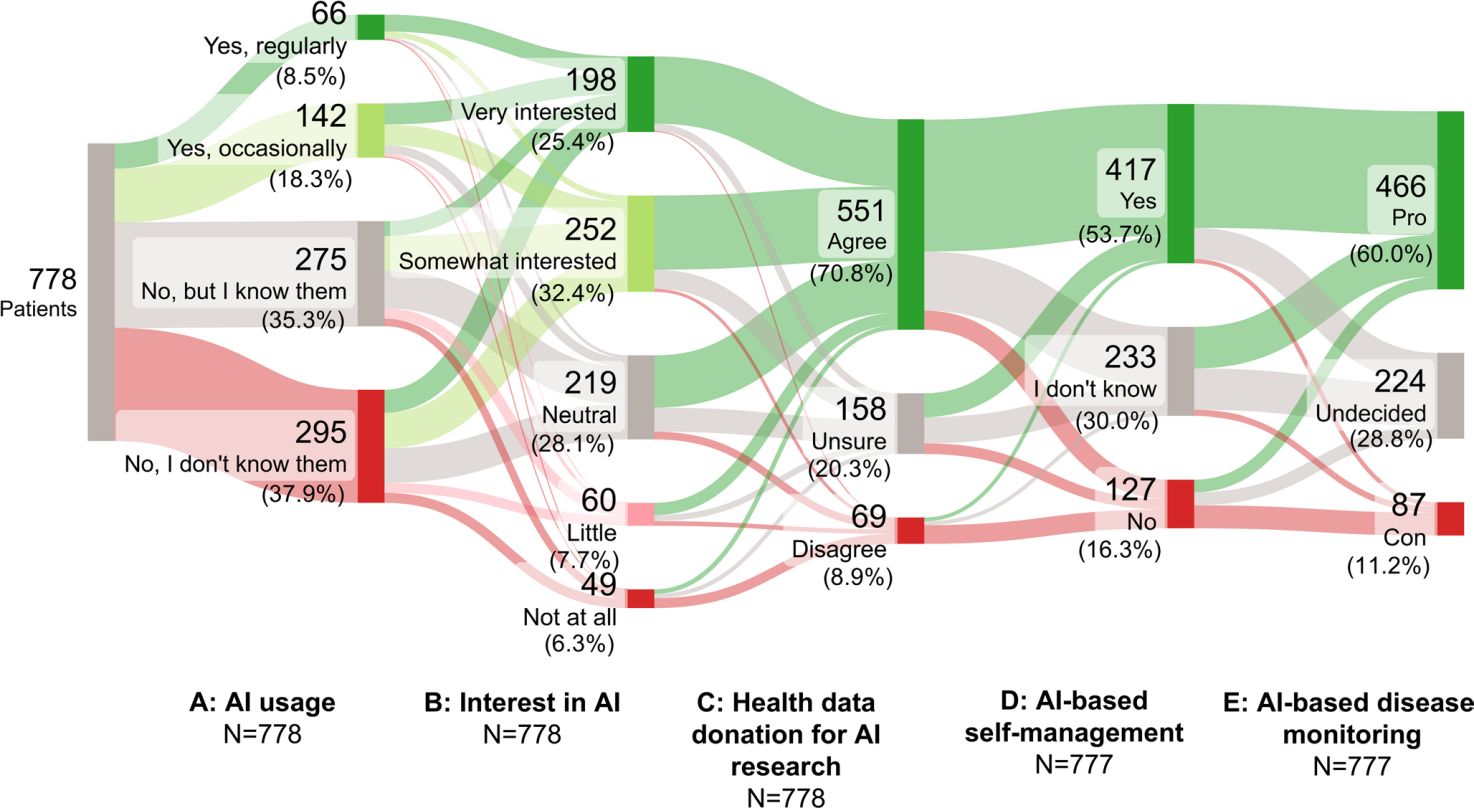
Sankey diagram to illustrate **A** usage of AI-based tools for health-related use cases, **B** interest in AI and willingness to **C** donate data for AI research, **D** use AI-based self-management and **E** employ AI-based disease monitoring using AI and wearables. (*N* = 777–778). The discrepancy in the sample size is due to one missing answer. Green pathways show positive responses, red shows negative, and grey shows neutral or undecided responses. The Sankey diagram was created using the SankeyMATIC online tool. Pro and con in Fig. 1E represent favorable and unfavorable attitudes toward AI-based disease monitoring

Among all respondents, 26.8% reported having used AI for health-related purposes, including 8.5% who used it regularly and 18.3% occasionally (see Fig. 1A). A total of 37.9% stated that they were not familiar with AI. Interest in AI rheumatology applications was expressed by 57.8% of patients (see Fig. 1B). Furthermore, 70.8% indicated a willingness to donate anonymized health data for AI research conducted by university hospitals (see Fig. 1C). More than half of the patients (53.7%) were interested in an AI-based platform to help better manage their rheumatic condition (see Fig. 1D). In addition, 60.0% agreed that their rheumatologist could use AI and wearables to monitor and analyze the course of their disease (see Fig. 1E).

### Current and Potential Use cases of AI in Rheumatology

**Fig. 2:**
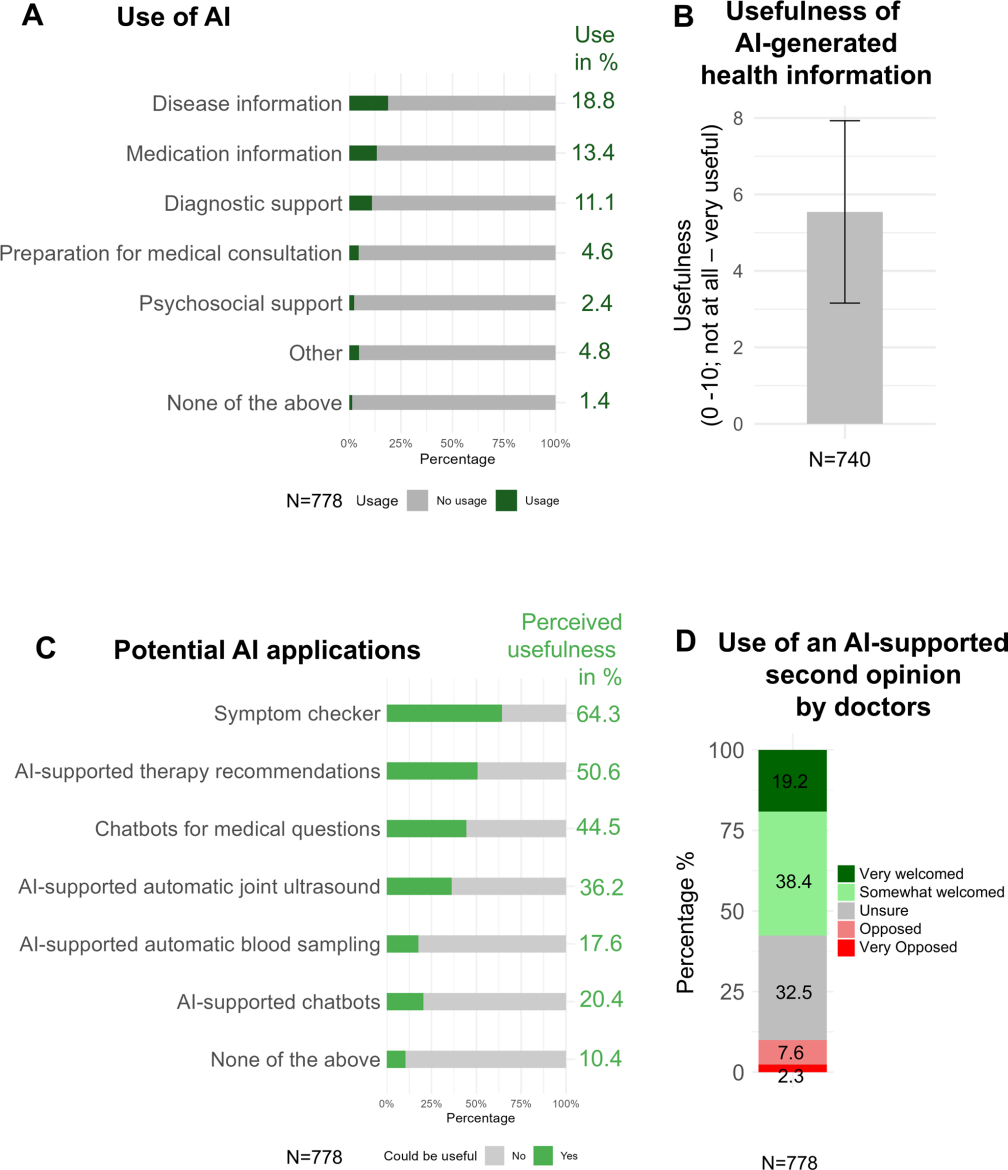
Current and Potential use of AI in healthcare and patient attitudes toward AI-assisted care. **A** shows in green how often patients currently use AI for specific healthcare purposes (N = 778). **B** shows the average perceived usefulness (mean ± SD) of AI among patients (N = 740). **C** shows the percentage of patients in light green who think that specifically defined rheumatology AI tools could be useful (N = 778). AI-supported automatic joint ultrasound’, ‘AI-supported automatic blood sampling’, and ‘AI-supported chatbot’ refer to prototype systems demonstrating artificial intelligence applications in musculoskeletal diagnostics, automated phlebotomy, and patient communication, respectively. **D** shows patients’ opinion on the use of AI-supported second opinion by physicians (*N* = 778).The figure was created using R and RStudio

The most commonly reported current use cases of AI included obtaining information about the disease (18.4%), accessing medication-related information (13.4%), and receiving diagnostic support (11.1%) (Fig. 2A). The perceived usefulness of AI-generated health information was rated with a mean (SD) score of 5.5 (2.4) on an 11-point Likert scale (0–10; not at all – very useful) (Fig. 2B). Regarding future applications, patients expressed the greatest interest in an AI-based symptom checker (64.3%), followed by AI-assisted therapy recommendations (50.6%) and AI-based chatbots for medical inquiries (44.5%) (Fig. 2C). Additionally, 57.6% of respondents indicated that they would (very much) welcome the use of AI-based second opinions by physicians for diagnostic and therapeutic decision-making (Fig. 2D). 10.4% of the patients rejected all the proposed potential applications of AI.

### Perceived AI Advantages, Barriers and Facilitators

**Fig. 3:**
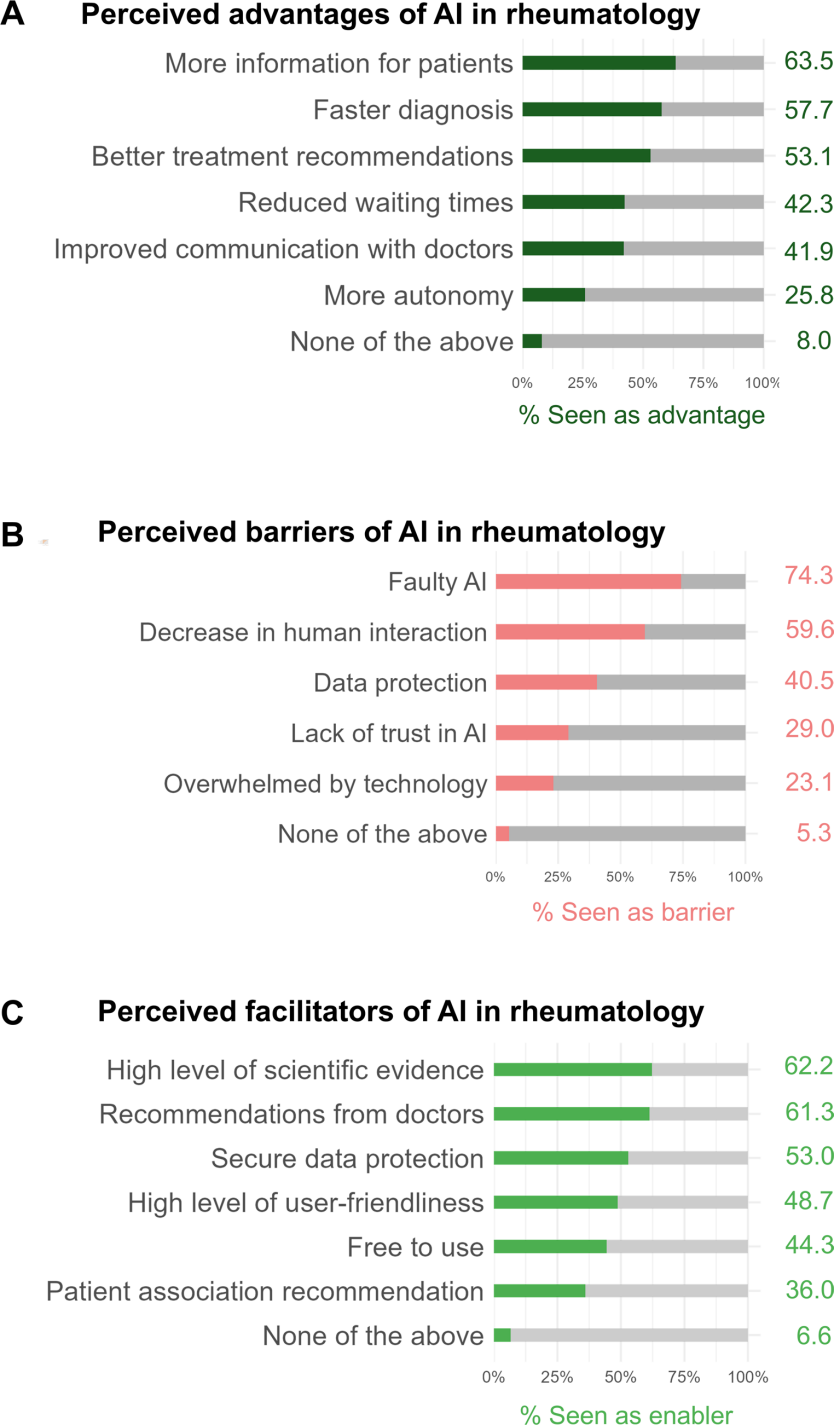
Perceived advantages, barriers and facilitators of AI in rheumatology. **A** shows the proportion of patients who recognize specific advantages of AI. **B** illustrates key concerns hindering AI adoption. **C** presents factors that could encourage AI use. Each stacked bar reflects the percentage of respondents who selected each item (*N* = 778). The figure was created using R and RStudio. The item “improved communication with doctors” was not further specified (e.g. frequency, quality, or efficiency) in the survey

The most commonly cited advantages were more information (63.5%), faster diagnosis (57.7%), and better treatment recommendations (53.1%), see Fig. 3A. Other noted benefits included reduced waiting times (42.3%), improved communication with doctors (41.9%), and greater patient independence (25.8%). Only a small portion (8.0%) indicated that none of the listed advantages applied.

The most frequently mentioned barriers were concerns about faulty AI (74.3%) and a decrease in human interaction (59.6%), see Fig. 3B. Further concerns included data protection issues (40.5%), lack of trust in AI (29.0%), and feeling overwhelmed by technology (23.1%). Only 5.3% of participants stated that none of the mentioned barriers were relevant to them.

Key facilitators of AI were a high level of scientific evidence (62.2%) and active recommendations for AI applications from doctors (61.3%), see Fig. 3C. Additional important facilitators included secure data protection (53.0%), high level of user-friendliness (48.7%), and free AI software (44.3%). Recommendations by patient associations were also cited (36.0%). Only 6.6% of respondents reported that none of the proposed facilitators would influence their acceptance of AI.

### Patient Clusters

**Fig. 4:**
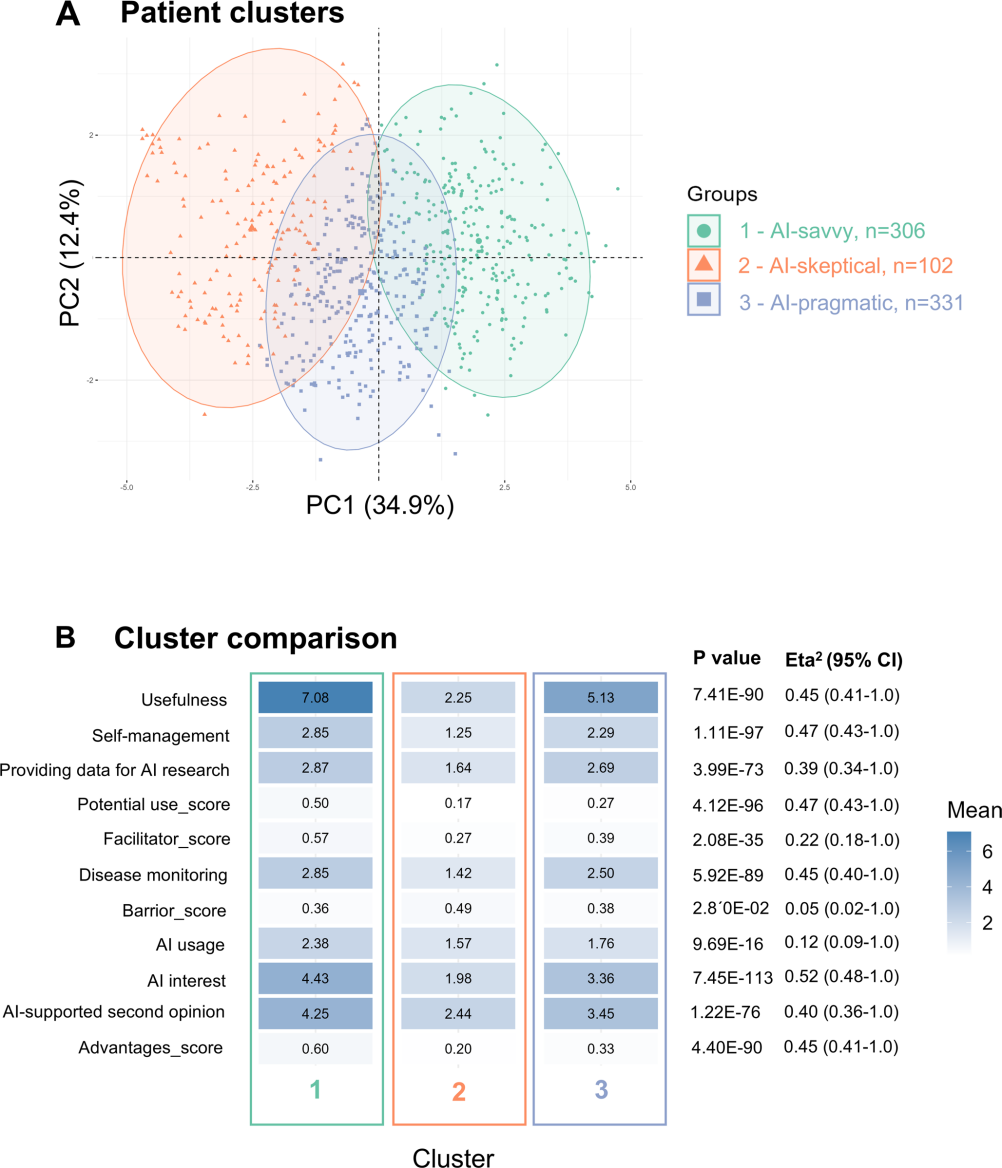
**A** Patient clusters and **B** their respective characteristics. By clustering analysis, three distinct patient groups based on their use of and attitudes toward AI in healthcare were identified. **A** shows clustering via Principal Component Analysis (PCA) scatterplot which illustrates the spatial distribution of patients across two principal dimensions derived from the data. Three distinct clusters are identified and visually separated using colored ellipses: cluster 1 – AI-savvy (*n* = 306): green, cluster 2 – AI-skeptical (*n* = 102): orange, cluster 3 – AI-pragmatic (*n* = 331): blue. **B** The heatmap shows differences between the three clusters across multiple variables by mean scores. Significant differences are indicated by p-values and confidence intervals (CI), *N* = 739. The graphs were created using R and RStudio

By cluster analysis, three patient clusters were identified with partially overlapping confidence ellipses in the PCA plot (Fig. 4A). Clusters 1 and 3 and Cluster 2 and 3 show some overlap, suggesting shared characteristics, whereas Cluster 1 and 2 are distinctly separated—particularly along the first principal component axis—indicating a greater divergence in attitudes and experiences related to AI use in rheumatology.

The clusters were further characterized by analyzing and statistically comparing the mean values of the variables included in the clustering (Fig. 4B). Cluster 1 (*n* = 306, 41.4%), labeled “AI-savvy,” consistently reported higher mean scores across variables such as AI interest, AI usage for health-related use cases, and interest in AI-supported second opinions. In contrast, Cluster 2 (“AI-skeptical,” *n* = 102, 13.8%) showed the lowest scores across nearly all domains, particularly for AI interest and potential use. Cluster 3 (“AI-pragmatic,” *n* = 331, 44.8%) occupied intermediate positions but displayed similarities with both of the other groups. All dimensions showed statistically significant differences between clusters with large effect sizes, especially for AI interest (η² = 0.52), AI-based self-management (η² = 0.47) and the assessment of potential AI applications (η² = 0.47). Only the perceived barriers and AI usage showed weaker effects (η² = 0.02 and 0.12, respectively), suggesting more uniform perceptions across groups for those variables.

The associations between cluster membership with demographic variables and rheumatic disease were assessed using Fisher’s exact tests. No statistically significant associations were observed for sex (*p* = 0.087), healthcare setting (*p* = 0.290), or type of rheumatic disease (*p* = 0.97), data not shown.

**Fig. 5:**
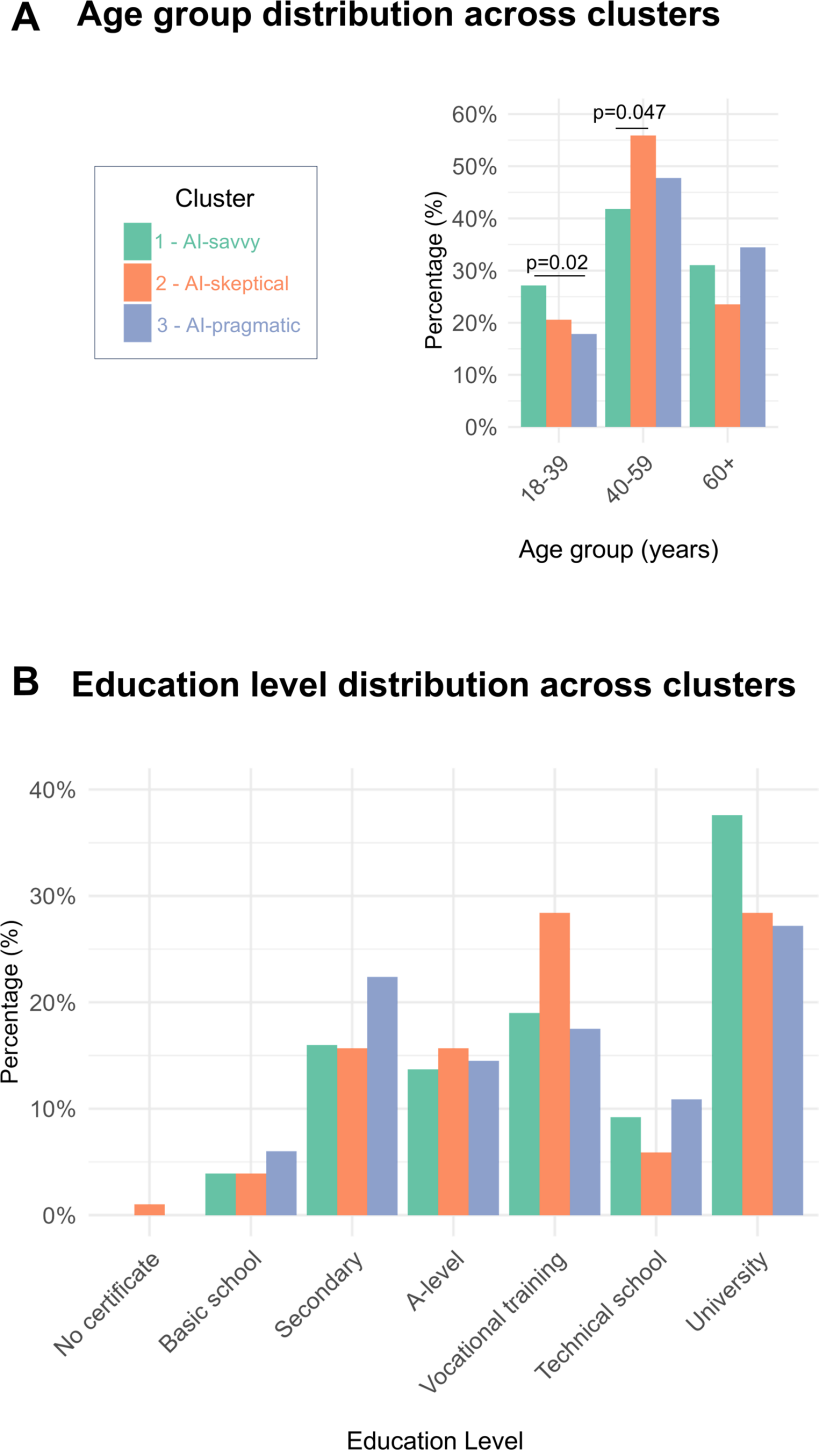
Age and education level distribution across clusters. **A** shows the figure legend. **B** displays the percentage of individuals in each cluster—AI-savvy (green), AI-skeptical (orange), and AI-pragmatic (blue)—across the three age groups: 18–39, 40–59, and 60 + years. **C** shows how education levels are distributed within each cluster, ranging from no certificate to university degree. Statistically significant differences (*p* < 0.05) between clusters are indicated, *N* = 739. The graphs were created using R and RStudio

However, significant differences were found for age groups (*p* = 0.012, Cramer’s V 0.33) and education levels (*p* = 0.045, Cramer’s V 0.13), see Fig. 5A and B. Specifically, a higher proportion of younger participants (aged 18–39 years, 27.1%) was observed in the “AI-savvy” group compared to the “AI-pragmatic” group (*p* = 0.02, 17.6%), while participants aged 40–59 years were more frequently represented in the “AI-skeptical” group compared to the “AI-savvy” group (*p* = 0.047, 55.9 vs. 41.8%), see Fig. 5B. Participants in the “AI-savvy” cluster were more likely to hold a university degree (37.6%), while those in the “AI-skeptical” cluster had a higher proportion of individuals with vocational training (28.4%), although pairwise Fisher’s exact tests did not reach statistical significance for these specific comparisons (Fig. 5B).

The statistical evaluation of individual survey items across the entire sample identified age as the most influential factor, showing significant associations with nearly all survey items, e.g. AI interest, AI usage and perceived usefulness with a moderate effect on AI usage (η² = 0.11, other η² between 0.01 und 0.06), see Supplementary file 2. In addition, significant differences were observed with respect to education level and AI interest and education level and current AI use Current AI use was significantly lower in patients with fibromyalgia (FM) (*p* = 0.003, η² = 0.11), with post-hoc comparisons revealing significant differences when compared to patients with axial spondyloarthritis (axSpA, *p* = 0.02) and SLE (*p* = 0.02), not explained by age differences between the respective groups (*p* = 0.6, 95% CI -3.6-6.1 and *p* = 0.7, 95% CI -5.7-3.7, respectively).

## Discussion

This study presents novel findings from the first national patient survey that systematically explores the use, perceptions, and expectations of AI among individuals with rheumatic diseases. These findings complement recent reviews [8] of AI use cases and developments in rheumatology and add the patient perspective to clinician-focused studies, such as the 2024 national survey of German rheumatologists conducted by Holzer et al. [7].

Notably, 27% of both patients and rheumatologists [7] reported using AI for health-related purposes. Consistent with earlier studies identifying information access as a top priority among patients [11, 12], AI in our survey was most commonly used to obtain health-related information. This aligns with data showing that only 24% of German rheumatology patients feel well-informed about their disease [13], highlighting the potential of AI to support patient education. Tools like www.lupusgpt.org, which use retrieval-augmented generation, are increasingly adopted and may offer information that is not only accessible but also comparable in quality to rheumatologists [5, 14].

Patients also showed strong interest in AI-supported diagnostics and endorsed its use by physicians for second opinions. Patients increasingly consult symptom checkers or online sources before medical appointments [12, 15]. These behaviors, along with persistent diagnostic delays [16], suggest opportunities for AI to improve early detection and referral. Recent studies show that off-the-shelf LLMs outperform existing decision support systems in diagnostic accuracy and speed [3]. A randomized controlled trial further demonstrated that medical students performed significantly better solving rheumatology vignettes when supported by ChatGPT [17]. While further research is needed, rheumatology may soon follow other specialties, such as oncology, where AI-supported screening is already routine.

In our study, the majority of rheumatology patients supported the use of AI-generated second opinions by doctors consistent with the results of a study involving breast cancer patients [18]. However, the findings from Reis et al. [19], which indicated that the public perceives fictional doctors using AI as less competent, less trustworthy, and less empathetic, seem to contradict our results.

Our exploratory clustering approach confirmed that the vast majority of patients show a certain affinity for AI and are at least not opposed to its future use in rheumatology, which is consistent with data on the German general population [20] and with results of a multinational survey on attitudes toward AI among hospital patients [21]. Only 102/739 (13.8%) patients clustered in the AI-skeptical group. More experience with AI in a medical context is likely to change patients’ perspectives in the future. The fact that 37.9% of study participants stated they “did not know about AI” is an important finding of our study, clearly demonstrating the need for education.

The identification of three distinct patient clusters confirms the heterogeneity of AI attitudes. In line with previous findings on digital health literacy [11], younger and more educated patients were more likely to belong to the AI-savvy group. This finding that age and education are linked to AI attitudes is important, as it suggests that implementation strategies should account for these demographic differences. Specifically, tailored patient education and communication strategies may be needed to engage older or less educated patient groups and ensure equitable understanding and acceptance of AI-based health technologies.

Patients with fibromyalgia reported lower current use of AI compared to those with other rheumatic diseases. The underlying reasons for this observation warrant further investigation in larger, dedicated studies, given the small sample size and modest effect. When implementing AI in rheumatology, it will be crucial to pay special attention to these groups to prevent further exacerbation of health inequalities.

Although patients perceived multiple benefits, they also voiced concerns about data privacy, AI reliability, and reduced human interaction. The fact that nearly three-quarters identified faulty AI as a major barrier underlines the importance of ensuring transparency, reliability, and clinical validation of AI systems before their widespread implementation. Concerns about reduced human interaction suggest that patients value the interpersonal aspects of care and may resist AI tools perceived as replacing, rather than supporting, clinicians. Furthermore, the notable proportion citing data privacy and trust issues emphasizes the need for clear communication about data protection, algorithmic accountability, and the complementary role of AI in shared decision-making. These concerns also proved to be significant in other studies involving non-rheumatological study participants [22–24]. Addressing these issues through transparent information, patient involvement in AI development, and clinician–patient dialogue will be crucial for fostering trust and acceptance. Scientific validation, physician endorsement, secure data protection and user-friendliness were key factors influencing acceptance, underscoring the important role of clinicians and academic institutions in guiding responsible AI integration. The finding that over one-third of patients regarded recommendations from patient organizations as a facilitator shows the critical role of such organizations when implementing AI in rheumatology.

A major strength of this study is its multicenter design, including university and non-university rheumatology centers and a broad range of rheumatic diseases. The direct involvement of patient representatives throughout all study phases further strengthens the relevance of the findings. Nonetheless, the online format and QR code access likely introduced a considerable selection bias, favoring digitally engaged and higher-educated individuals which may have biased the results toward greater AI acceptance. Additionally, as the survey was conducted in Germany, the findings reflect the country’s specific digitalization culture, which may limit their generalizability to other healthcare systems. Patients with a university degree were slightly overrepresented compared to data on the educational level of the general German population [25], which may have influenced representativeness of the results. A further methodological limitation is the use of self-reported diagnoses.

PCA and cluster analysis were employed to identify and visualize patient clusters representing distinct attitudes and experiences related to AI use. Both techniques are widely applied for dimensionality reduction and pattern identification, yet they entail certain methodological limitations. PCA assumes linear relationships among variables and may result in information loss during dimensionality reduction. As with most clustering methods, the cluster analysis results depend on the type and number of variables included, meaning that changes in variable selection can produce different cluster solutions, even within the same dataset. Despite these constraints, PCA provides an efficient means of summarizing complex datasets while retaining most of their variance, and cluster analysis reveals latent group structures that enhance and clarify data interpretation.

## Conclusion

This study highlights a clear gap between patients’ current use of AI for health-related purposes and their interest in AI-supported medical applications. Patients and rheumatologists share similar priorities, particularly around diagnostics and education. Realizing the full potential of AI in rheumatology will require rigorous validation, targeted implementation strategies, and a proactive role from the rheumatology community in ensuring that AI serves both clinical and patient needs effectively.

## Supplementary Information

Below is the link to the electronic supplementary material.

Supplementary Material 1

Supplementary Material 2

## Data Availability

The raw data supporting the conclusions of this article will be made available by the authors upon reasonable request.
