# Supplementary material for: Patient experiences, attitudes, and profiles regarding artificial intelligence in rheumatology: a German national cross-sectional survey study
Source: Rheumatol Int. 2025 Nov 10;45(12):269. doi: 10.1007/s00296-025-06023-x (PMC12602644; doi:10.1007/s00296-025-06023-x)
Supplement: Supplementary file 1 — Supplementary Material 1 [file 296_2025_6023_MOESM1_ESM.docx]

Survey on Artificial Intelligence in Rheumatology

Thank you very much for supporting us by completing this short survey.

Gender:

Female
Male
Other

Highest level of education:

No degree
Lower secondary school
Intermediate secondary school
(Technical) high school diploma
Vocational training
Technical college (e.g., master craftsman)
University degree
Other

Age: __________________________________ (Years, e.g., 35)

Where are you mainly treated for your rheumatic condition?

University hospital
Other hospital
Private practice

Rheumatic disease:

Rheumatoid arthritis
Psoriatic arthritis
Axial spondyloarthritis
Gout
Lupus
Myositis
Polymyalgia rheumatica (PMR)
Polyosteoarthritis
Giant cell arteritis
Scleroderma
Other disease

Please name the disease:
__________________________________

How interested are you in AI-based applications in rheumatology?

Not at all
Slightly
Neutral
Rather interested
Very interested

Have you already used AI-based tools or chatbots (e.g., ChatGPT, Ada Health) for health-related questions?

Yes, regularly
Yes, occasionally
No, but I’m aware of them
No, I’m not aware of them

For what purpose have you used AI?

Information about my disease
Medication information
Diagnostic support/symptom clarification
Preparation for doctor visits
Psychosocial support
Other
None of the above

Please describe your usage:
__________________________________

How helpful do you find AI-generated health information?
(Not helpful – Very helpful)

How high do you estimate the risk of AI providing misinformation for health-related topics?

Very low
Low
Medium
High
Very high

How do you feel about doctors using AI-based second opinions (with data protection) to improve your treatment?

Would strongly welcome
Would rather welcome
Neutral
Rather opposed
Strongly opposed

Which types of AI-based applications would you most likely use? (Multiple answers possible)

Symptom checkers (to identify and interpret symptoms)
Medical chatbots
AI-supported therapy recommendations
AI-assisted automated joint ultrasound at the doctor’s office
AI-assisted automated blood sampling at the doctor’s office
AI-supported chatbots for psychosocial support and patient education
None of the above

Which benefits do you expect from AI in rheumatology? (Multiple answers possible)

Faster diagnosis
Better therapy recommendations
More information for patients
More independent management
Improved communication with doctors
Shorter waiting times
None of the above
(Please select at least one answer)

What concerns do you have regarding AI in medicine? (Multiple answers possible)

Data protection
Faulty AI
Decreased human interaction
Lack of trust in AI
Overwhelmed by technology
None of the above
(Please select at least one answer)

Would you be willing to make your health data available anonymously for AI research by university hospitals in rheumatology?

Yes
No
Don’t know

Would you be interested in an AI-assisted platform to better manage your condition?

Yes
No
Don’t know

Would you agree to your doctors using AI to monitor/analyze disease progression through smart devices (e.g., wearables)?

Yes
No
Don’t know

Which factors would most influence your decision to use AI-based services?

Recommendations from doctors
Recommendation from the Rheumatism League
High level of scientific evidence
Guaranteed data protection
High user-friendliness
Free of charge
None of the above
