# Supplementary material for: Patient experiences, attitudes, and profiles regarding artificial intelligence in rheumatology: a German national cross-sectional survey study
Source: Rheumatol Int. 2025 Nov 10;45(12):269. doi: 10.1007/s00296-025-06023-x (PMC12602644; doi:10.1007/s00296-025-06023-x)
Supplement: Supplementary file 2 — Supplementary Material 2 [file 296_2025_6023_MOESM2_ESM.docx]

**Factors associated with survey responses**
Demographic categories, subcategories with group sizes n, mean and SD of the individual survey items (questions 10+11, questions 14-16) are presented and differences are statistically evaluated.
*Abbreviations: VAS, visual analogue scale; SD, standard deviation; d, Cohen’s d; η², Eta squared; RA, rheumatoid arthritis; PsA, psoriatic arthritis; axSpA, axial spondyloarthritis; SLE, systemic lupus erythematosus; CTD, connective tissue disease; OA, osteoarthritis; FM, fibromyalgia.*

| Category | Subcategory | N  (Q10,Q11) | Q10: AI interest  *(1 Not at all,  2 Not very,*  *3 Neutral,  4 Rather interested,  5 Very interested)* | | Q11: AI usage *(1 Yes, regularly*  *2 Yes, occasionally*  *3 No, but I know them*  *4 No, I don't know them)* | | N  (Q14) | Q14: AI medical usefulness  *(VAS 0-10;*  *0 Not at all,*  *10 Very)* | |
| --- | --- | --- | --- | --- | --- | --- | --- | --- | --- |
|  | | | Mean (SD) | P value,  *Effect size* *(*Post-hoc p value) | Mean  (SD) | P value,  *Effect size* *(*Post-hoc p value) |  | Mean  (SD) | P value,  *Effect size* *(*Post-hoc p value) |
| Total n | -- | 778 | 3.6 (1.1) | -- | 3.0 (0.9) | -- | 740 | 5.5 (2.4) | -- |
| Gender | Female | 548 | 3.6 (1.1) | 0.05 *d=-0.15* | 3.1 (0.9) | 0.07,  *d=0.14* | 521 | 5.4 (2.4) | **0.03** *d=-0.17* |
|  | Male | 229 | 3.8 (1.1) |  | 2.9 (1.0) |  | 218 | 5.8 (2.3) |  |
| Age (years) | A: 18-39 | 172 | 3.7 (1.1) | **0.005,** *η²=0.014* *(*B vs. C: 0.003*)* | 2.5 (1.0) | **<0.001,** *η²=0.11* (A vs. B,  A vs. C: <0.001;   B vs. C:   0.02) | 163 | 6.0 (2.2) | **<0.001** *η²=0.03*  (A vs. B;  B vs. C: <0.001) |
|  | B: 40-59 | 359 | 3.5 (1.2) |  | 3.1 (0.9) |  | 344 | 5.1 (2.5) |  |
|  | C: ≥ 60 | 246 | 3.8 (1.1) |  | 3.3 (0.8) |  | 233 | 5.9 (2.3) |  |
| Education | A: No school-leaving certificate | 2 | 3.0 (2.8) | **0.01,** *η²=0.02*  (E vs. G: 0.02) | 4.0 (0) | **<0.001,**  *η²=0.04*  (G vs. B:  0.04,  G vs. C:  0.0002,  G vs. F:  0.01) | N/A | N/A | 0.3 *η²=0.01* |
|  | B: Basic secondary school certificate | 41 | 3.3 (1.2) |  | 3.3 (1.0) |  | 36 | 5.2 (2.6) |  |
|  | C: Secondary school certificate | 148 | 3.6 (1.0) |  | 3.3 (0.8) |  | 139 | 5.4 (2.2) |  |
|  | D: German A-level equivalent | 109 | 3.6 (1.1) |  | 3.0 (1.0) |  | 106 | 5.5 (2.4) |  |
|  | E: Vocational training | 149 | 3.4 (1.3) |  | 3.0 (1.0) |  | 145 | 5.6 (2.6) |  |
|  | F: Technical school | 79 | 3.6 (1.0) |  | 3.2 (0.8) |  | 70 | 5.6 (2.0) |  |
|  | G: University degree | 240 | 3.8 (1.1) |  | 2.8 (1.0) |  | 235 | 5.7 (2.3) |  |
|  | H: Other | 10 | 3.9 (1.3) |  | 3.1 (1.0) |  | 8 | 5.0 (2.9) |  |
| Treating center | A: University hospital | 301 | 3.7 (1.1) | 0.7 | 3.0 (1.0) | **0.01,**  *η²=0.01*  (B vs.C: 0.01) | 169 | 5.7 (2.4) | 0.06 *η²=0.007* |
|  | B: General hospital | 178 | 3.7 (1.1) |  | 2.9 (1.0) |  | 287 | 5.7 (2.3) |  |
|  | C: Outpatient practice | 297 | 3.6 (1.2) |  | 3.1 (0.9) |  | 283 | 5.3 (2.4) |  |
| Disease | A: RA | 247 | 3.7 (1.1) | 0.9 | 3.1 (0.9) | **0.003,**  *η²=0.03* (I vs. C ,   I vs. D :  0.02 ;  I vs. J:  0.01) | 239 | 5.5 (2.4) | 0.7 *η²=0.009* |
|  | B: PsA | 99 | 3.6 (1.1) |  | 3.0 (0.9) |  | 92 | 5.2 (2.3) |  |
|  | C: axSpA | 79 | 3.7 (1.1) |  | 2.8 (1.1) |  | 76 | 5.7 (2.2) |  |
|  | D: SLE | 78 | 3.7 (1.1) |  | 2.8 (1.0) |  | 74 | 6.0 (2.2) |  |
|  | E:Vasculitis | 57 | 3.4 (1.1) |  | 3.2 (0.9) |  | 57 | 5.7 (2.5) |  |
|  | F: Myositis | 53 | 3.7 (1.1) |  | 3.0 (1.0) |  | 51 | 5.4 (2.5) |  |
|  | G: Other CTD | 52 | 3.8 (0.9) |  | 3.0 (1.0) |  | 47 | 5.6 (2.1) |  |
|  | H: OA | 18 | 3.4 (1.2) |  | 3.3 (0.8) |  | 18 | 5.7 (2.2) |  |
|  | I: FM | 17 | 3.6 (1.3) |  | 3.7 (0.5) |  | 17 | 5.0 (3.5) |  |
|  | J: Other | 68 | 3.6 (1.3) |  | 2.8 (0.9) |  | 62 | 5.8 (2.3) |  |

| Category | Subcategory | N  Q15+16 | Q15: AI risk of misinformation *(1 Very low; 2 Low; 3 Medium;*  *4 High; 5 Very high)* | | Q16: AI use by doctors *(1 I would very much welcome;  2 I would rather welcome;  3 Neutral;*  *4 I am rather opposed;*  *5 I am very opposed)* | |
| --- | --- | --- | --- | --- | --- | --- |
|  | | | Mean (SD) | P value,  *Effect size* *(*Post-hoc p value) | Mean (SD) | P value,  *Effect size* *(*Post-hoc p value) |
| Total n | -- | 778 | 3.1 (0.8) | -- | 2.4 (1.0) | -- |
| Gender | Female | 548 | 3.1 (0.8) | 0.2 *d=0.1* | 2.4 (1.0) | **0.001** *d=0.24* |
|  | Male | 229 | 3.0 (0.9) |  | 2.2 (0.9) |  |
| Age (years) | A: 18-39 | 172 | 3.2 (0.8) | **0.003** *η²=0.015*  (A vs.C:  0.006;  B vs. C: 0.02) | 2.4 (1.0) | **0.001**  *η²=0.017*  (B vs. C: 0.0007) |
|  | B: 40-59 | 359 | 3.1 (0.8) |  | 2.5 (1.0) |  |
|  | C: ≥ 60 | 246 | 2.9 (0.8) |  | 2.2 (0.9) |  |
| Education | A: No school-leaving certificate | 2 | 2.0 (1.4) | 0.1 *η²=0.015* | 2.0 (1.4) | 0.04  *η²=0.019*  (Post-hoc tests NS) |
|  | B: Basic secondary school certificate | 41 | 3.0 (0.6) |  | 2.5 (0.9) |  |
|  | C: Secondary school certificate | 148 | 2.9 (0.8) |  | 2.4 (0.9) |  |
|  | D: German A-level equivalent | 109 | 3.1 (0.8) |  | 2.4 (0.9) |  |
|  | E: Vocational training | 149 | 3.1 (0.9) |  | 2.5 (1.0) |  |
|  | F: Technical school | 79 | 3.1 (0.8) |  | 2.4 (0.9) |  |
|  | G: University degree | 240 | 3.2 (0.9) |  | 2.2 (1.0) |  |
|  | H: Other | 10 | 3.2 (1.1) |  | 2.9 (0.9) |  |
| Treating center | A: University hospital | 178 | 3.1 (0.8) | 0.7 *η²=0.0009* | 2.3 (0.9) | **0.003** *η²=0.015*  (B vs. C:  0.005;  A vs. C: 0.02) |
|  | B: General hospital | 297 | 3.1 (0.8) |  | 2.2 (1.0) |  |
|  | C: Outpatient practice | 301 | 3.1 (0.8) |  | 2.5 (1.0) |  |
| Disease | A: RA | 247 | 3.1 (0.8) | 0.3  *η²=0.015* | 2.4 (1.0) | 0.8 |
|  | B: PsA | 99 | 3.1 (0.8) |  | 2.3 (1.0) |  |
|  | C: axSpA | 79 | 3.0 (0.9) |  | 2.4 (0.9) |  |
|  | D: SLE | 78 | 2.9 (0.8) |  | 2.4 (0.9) |  |
|  | E: Vasculitis | 57 | 2.9 (0.9) |  | 2.2 (1.0) |  |
|  | F: Myositis | 53 | 3.1 (0.8) |  | 2.2 (1.1) |  |
|  | G: Other CTD | 52 | 3.3 (0.7) |  | 2.3 (0.8) |  |
|  | H: OA | 18 | 3.2 (0.6) |  | 2.3 (0.8) |  |
|  | I: FM | 17 | 2.9 (1.0) |  | 2.4 (1.2) |  |
|  | J: Other | 68 | 3.2 (0.9) |  | 2.4 (1.0) |  |
